# Optimizing Agronomic Zinc Biofortification in Carrots

**DOI:** 10.1002/pei3.70086

**Published:** 2025-09-23

**Authors:** Godfred Okyere‐Prah, Paul A. Asare, Kwadwo K. Amoah, Kofi Atiah, Moses Teye, Alfred A. Darkwa, Roseline L. Macarthur, Michael O. Adu

**Affiliations:** ^1^ Department of Crop Science, School of Agriculture, College of Agriculture and Natural Sciences University of Cape Coast Cape Coast Ghana; ^2^ Department of Soil Science, School of Agriculture, College of Agriculture and Natural Sciences University of Cape Coast Cape Coast Ghana; ^3^ Department of Animal Science, School of Agriculture, College of Agriculture and Natural Sciences University of Cape Coast Cape Coast Ghana; ^4^ Department of Vocational and Technical Education, Faculty of Science and Technology Studies, College of Education Studies University of Cape Coast Cape Coast Ghana

**Keywords:** *Daucus carota* L, nutrient accumulation, time of Zn application, tropical agriculture, Zn application methods, Zn biofortification

## Abstract

Zn deficiency affects over 2 billion people globally, particularly in regions with Zn‐deficient soils. Biofortification of staple crops offers a sustainable solution to this challenge. This study investigated Zn application methods (soil vs. foliar), rates (0–6 kg ha^−1^), and timings (30, 50, and 70 days after sowing [DAS]) on growth, yield, and Zn accumulation in carrots under greenhouse conditions. Zn application significantly improved plant growth parameters, chlorophyll content, and yield. Chlorophyll content increased by approximately 36% as Zn rates increased from 0 to 6 kg ha^−1^. Zn application at 6 kg ha^−1^ increased carrot yield by 97.2% compared to the control. Foliar application achieved superior shoot Zn enrichment, with concentrations 51% higher than soil application at the highest rate. Root Zn concentrations showed no significant difference between application methods, suggesting distinct Zn translocation mechanisms between aerial and underground tissues. Early application (30 DAS) was most effective for root Zn accumulation, increasing concentrations by 175% compared to the control. An observed quadratic response to Zn application suggests an optimal threshold (6 kg ha^−1^) for maximizing biofortification efficiency while maintaining economic feasibility. Early Zn intervention is essential for sandy soils with rapid nutrient leaching potential, and combined soil–foliar applications effectively address limited nutrient retention capacity. These findings demonstrate that Zn biofortification can simultaneously meet nutritional objectives and improve agricultural productivity in carrots, providing viable strategies for regions with similar soil constraints.

## Graphical abstract

Through optimized application methods, rates, and timing, zinc biofortification in carrots significantly enhances growth, yield, and nutrient accumulation. Foliar application at 6 kg ha^−1^ zinc increased shoot zinc concentration by 51% compared to soil application, while early application (30 days after sowing) boosted root zinc levels by 175%. The findings demonstrate the potential of an integrated zinc biofortification strategy to simultaneously address nutritional deficiencies and improve agricultural productivity in tropical regions with zinc‐deficient soils.

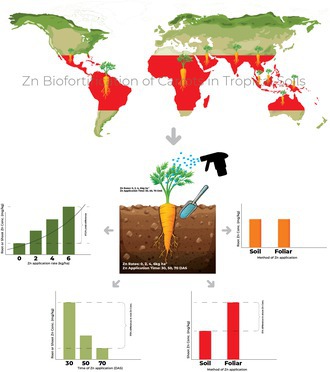

## 1 Introduction

Zinc (Zn) deficiency affects over two billion people worldwide (Hussain et al. 2022; Orbatu et al. 2025; Yokokawa et al. 2024), making it one of the most common micronutrient deficiencies (Chasapis et al. 2020; Hacisalihoglu 2020), particularly in developing regions where populations rely heavily on plant‐based diets. This micronutrient inadequacy leads to compromised immune function, impaired growth and development, increased susceptibility to various diseases, and, under extreme conditions, death, with pregnant women and children being particularly at risk (Chasapis et al. 2020). In developing countries, Zn deficiency is the fifth leading cause of disease and mortality. It is estimated that around 60%–70% of Sub‐Saharan African populations could suffer from Zn insufficiency due to an overdependence on cereal‐based diets and limited access to Zn‐rich foods (Ayan Das et al. 2019).

The prevalence of Zn deficiency is exacerbated by soil conditions in many affected regions. Soils from tropical regions, for instance, have high phosphorus (P) fixation, resulting in the need to use corrective phosphate fertilizers, which can induce Zn deficiency. Regions with soil Zn deficiency are deeply correlated with those with a high incidence of Zn deficiency (Floriano et al. 2018; Peramaiyan et al. 2022). This interconnection between soil health and human nutrition underscores the global complexity of addressing Zn deficiency. Biofortification, which enhances micronutrient content in staple crops through agronomic or genetic approaches, is a sustainable strategy to address this global health challenge (White and Broadley 2005). By targeting the root cause of Zn deficiency in soils and crops, biofortification offers a promising avenue for improving Zn nutrition in vulnerable populations while addressing the underlying agricultural factors contributing to this widespread micronutrient deficiency.

Root vegetables, particularly carrots ( *Daucus carota* L.), are important dietary sources of essential nutrients (Ahmad et al. 2019; Knez et al. 2022) and are widely produced and consumed globally (Motegaonkar et al. 2024). Carrots are cultivated on approximately 1.13 million ha and produce over 41 million tons globally (Buturi et al. 2023). While carrots are primarily recognized for their β‐carotene content, they may also possess significant potential for Zn biofortification due to their extensive tap root system and capacity for mineral accumulation (Knez et al. 2022). However, the efficiency of Zn uptake and translocation in carrots may be influenced by various factors, including the timing and method of Zn application, soil conditions, and plant developmental stages (Bhatt et al. 2020; Buturi et al. 2023). While both soil and foliar applications of Zn have shown promise in enriching crops, there is a lack of studies comparing these methods for carrot biofortification (Praharaj et al. 2021; Rugeles‐Reyes et al. 2019).

Previous research has established that foliar application of Zn can be an effective method for improving grain Zn concentration in cereals, with the timing of application being crucial for optimal results (Bhatt et al. 2020). Research on optimal Zn application methods for crop biofortification shows that combined application approaches generally outperform single methods. In rice, the combination of soil application, root dipping, and foliar spray (SA + RD + FS) produced the highest grain yields. In contrast, soil plus foliar application (SA + FS) maximized grain Zn content, though bioavailability remained below 1% (Das et al. 2019). For maize, seed treatment at 4 g kg^−1^ proved most cost‐effective and environmentally sustainable while enhancing Zn content (Ladumor et al. 2019). In chickpea, foliar application of Zn‐EDTA at three growth stages (vegetative, flowering, and grain‐filling) achieved superior grain yields and Zn biofortification compared to soil application, with crop recovery efficiency reaching 17.33% (Shivay et al. 2015). Wheat studies demonstrated that soil application of ZnSO_4_·7H_2_O as top dressing and foliar application combined with urea effectively increased grain Zn concentration while reducing phytic acid content (Akca and Taban 2024).

While both soil and foliar applications of Zn have shown promise in enriching crops, the comparative effectiveness of these methods in carrot biofortification remains poorly understood. The optimal timing and rate of Zn application for carrots are particularly unclear, as root vegetables' physiology and nutrient accumulation patterns differ significantly from extensively studied cereal crops. The temporal dynamics of Zn uptake and distribution in carrots during different growth stages, especially concerning application methods, require investigation. Understanding the impact of Zn application methods, rates, and timing is crucial for developing efficient biofortification protocols for carrots. Early intervention could influence root development and nutrient uptake efficiency, while application methods and timing may interact to affect Zn accumulation in edible portions. This study, therefore, aimed to optimize Zn biofortification strategies in carrots by investigating: (i) the effects of Zn application rates (0–6 kg ha^−1^) and timing [30, 50, and 70 days after sowing (DAS)] on Zn accumulation in shoots and edible roots; (ii) the comparative efficacy of soil versus foliar Zn application methods; and (iii) the interactions between application method, rate, and timing on Zn uptake, plant growth parameters, and yield components. We hypothesized that Zn biofortification efficiency in carrots will be optimized through strategic manipulation of application parameters, specifically: (i) foliar application will achieve superior tissue Zn enrichment compared to soil application; (ii) early Zn application will result in higher tissue Zn concentrations than later applications; and (iii) Zn application rates will impact tissue Zn concentration.

## 2 Materials and Methods

### 2.1 Overview of Environmental Conditions and Soil Preparation

A greenhouse experiment was conducted in the major rainy season of Ghana from May 2023 to August 2023 at the A.G. Carson Technology Centre of the School of Agriculture, University of Cape Coast (UCC) (5.1155° N, 1.2909° W) in the Central Region of Ghana. The study utilized soil collected from an arable site near the A.G. Carson Technology Centre of the University of Cape Coast at 0–0.15 m depth. The soil, characteristic of the coastal savanna region, was classified as a sandy loam with properties typical of Haplic Acrisols (IUSS Working Group World Reference Base 2015). Soil samples were collected from multiple locations at the site and composited samples after removing all plant debris. The composite samples were air‐dried for 3 days and were subsequently sieved using a 2 mm mesh. The resulting fine soil fraction (< 2 mm) was used for laboratory analysis and the pot experiment. Physicochemical analysis of the soil was done using standard laboratory methodologies. Texture was determined using the Bouyoucos hydrometer method (Bouyoucos 1962). The pH was measured in a 1:2.5 soil‐to‐water ratio using a glass electrode pH meter. Organic carbon was measured by the Walkley and Black (1934) wet oxidation method. Available phosphorus was extracted with Bray 1 solution (Bray & Kurtz, 1945), and CEC was determined using 1 N ammonium acetate at pH 7.0 (Rhoades 1982), after which the individual cations were summed up. The exchangeable calcium (Ca) and magnesium (Mg) were measured spectrometrically (Analyst 800, PerkinElmer, USA) with potassium (K) and sodium (Na) measurements done using a Jenway flame photometer (PFP7) (Jenway, Bibby Scientific Ltd., Staffordshire, UK). The texture of the soil was a sandy loam with 783.9 g kg^−1^ sand, 130 g kg^−1^ silt, and 83.2 g kg^−1^ clay. The soil exhibited a slightly acidic pH of 5.77 and a low organic matter content of 14.8 g kg−1. The cation exchange capacity (CEC) was 3.65 cmol kg^−1^, with calcium (2.01 cmol kg^−1^) and magnesium (1.18 cmol kg^−1^) being the dominant exchangeable cations. Available zinc content was 2.19 mg kg^−1^. The soil's carbon content was 8.6 g kg^−1^, and the exchangeable acidity was minimal at 0.09 cmol kg^−1^. Before planting, the soil was incubated at ambient temperature for 30 days after being moistened to 80% field capacity, determined gravimetrically. The experiment utilized nursery polybags with a volume of 10,996 cm^3^, equipped with drainage holes at the bottom. Each bag was filled with 18 kg of soil and carefully repacked to achieve a bulk density of 1.1 g cm^−3^.

### 2.2 Genetic Material, Experimental Design, Treatments, and Fertilizer Application

The experiment utilized “Kuroda king”, a widely cultivated carrot variety in Ghana with a maturation period of 85–95 days. Seeds were sourced from Sakaata Agro, a reputable agro‐input vendor in Accra, Ghana. A three‐factorial experiment was implemented using a Randomized Complete Block Design (RCBD) with three replications. The experimental factors were application method (soil and foliar), application rate (0, 2, 4, and 6 kg ha^−1^), and timing of application (30, 50, and 70 days after sowing [DAS]). This design resulted in 24 treatments, totaling 72 experimental units. Zinc application rates (0, 2, 4, and 6 kg ha^−1^) were selected based on literature baselines for vegetable biofortification and preliminary trials. Previous research indicated that optimal zinc rates for vegetable crops typically range from 5 to 30 kg ha^−1^ depending on application method and soil conditions, with tomatoes achieving maximum biofortification at 5 kg Zn ha^−1^ (Rabbi et al. 2024) and multiple vegetables including carrots showing optimal responses at 30 kg ZnSO_4_·7H_2_O ha^−1^ in alluvial soils (Solanki et al. 2010). Pre‐experiment trials confirmed that rates above 6 kg ha^−1^ showed diminishing returns in our sandy loam conditions, establishing this as the upper limit for the experimental range. Application timings (30, 50, and 70 DAS) were selected based on the maturity period of the Kuroda carrot cultivar, which reaches maturity at 80–90 days after sowing but can be harvested as early as 70 days. These intervals represent early vegetative growth (30 DAS), mid‐development (50 DAS), and near‐maturity/harvest stage (70 DAS), allowing evaluation of zinc uptake patterns from early development through the harvest‐ready stage. Zn sulfate heptahydrate (ZnSO_4_·7H_2_O) with a commercial purity of 99% (Sigma‐Aldrich, LabMart Knowledge Centre, Ghana) was the Zn source for soil and foliar applications. The appropriate Zn rate was dissolved in 100 mL of deionized water for each treatment. Soil applications involved direct administration of the Zn solution to the pot substrate. At the same time, foliar treatments were applied using a calibrated 2 L pump spray bottle to ensure uniform coverage of the plant canopy. Drift shields were employed during foliar applications to prevent cross‐contamination between treatments. Basal fertilization was standardized across all treatments to isolate the effects of Zn application. Nutrient rates applied 21 days after sowing included 222 kg ha^−1^ nitrogen (N) from urea (46% N), 133 kg ha^−1^ phosphorus (P_2_O_5_) from triple superphosphate, and 999 kg ha^−1^ potassium (K_2_O) from muriate of potash. These rates were based on recommendations from the Ministry of Food and Agriculture, Ghana (http://mofa.gov.gh/site/?page_id=14167; accessed 03/02/2023) and were scaled appropriately for pot‐based experiments.

### 2.3 Planting and Agronomic Practices

The carrot seeds were sown directly into the prepared pots at a depth of 1–2 cm and lightly covered with soil. The pots were temporarily mulched to protect the seeds from excessive heat and prevent displacement during watering. Germination occurred approximately 1 week after planting when the palm fronds were removed. Once established, the seedlings were thinned to maintain a spacing of approximately 3 cm between plants, resulting in 10 plants per pot. Irrigation was managed by maintaining soil moisture at 80% field capacity through daily watering with pipe water. Biweekly cultivation practices included gently stirring the soil between carrot rows using a hand fork to control weeds and improve soil aeration and water infiltration. The exposed upper portions of the developing carrot roots were periodically earthed up to prevent greening. All experimental units consistently applied these cultural practices to ensure uniform growing conditions.

### 2.4 Data Collection

Plant growth parameters and physiological measurements were recorded throughout the experiment. Three plants per pot were randomly selected and tagged for data collection. Plant height was measured using a wooden meter rule, while chlorophyll content was nondestructively assessed using a SPAD meter. These measurements were initiated 7 days after treatment imposition and continued biweekly until plant maturity. Harvest was conducted 90 days after planting. The carrots were carefully extracted, and the roots were thoroughly washed with tap water to remove adhering soil particles. Three plants per pot were randomly selected for analysis. These samples were rinsed with deionized water to eliminate potential contaminants and separated into aboveground and belowground biomass. Fresh weights of both shoot and root components were recorded immediately after separation. Root morphological characteristics were documented for the selected samples, including root diameter (measured 1 cm from the shoulder using digital vernier calipers) and root length (measured with a 30 cm ruler). The total yield was determined by weighing the roots of all 10 plants per pot.

The selected plant samples were then subjected to a standardized drying protocol. Shoot and root tissues were placed in separate labeled envelopes and oven‐dried at 70°C until a constant weight was achieved. The resulting dry weights were recorded for both shoot and root components. The dried samples were ground to a fine powder using a stainless‐steel laboratory mill to ensure homogeneity. The powdered samples were stored in airtight, contaminant‐free Ziploc bags at room temperature, pending nutritional analysis.

### 2.5 Determination of Tissue Zinc

Zinc concentration in carrot tissues was determined using a modified aqua regia digestion method using concentrated HCl and HNO_3_ (3:1 v/v) (Allen et al. 1974) followed by flame atomic absorption spectrophotometry (FAAS) analysis. The modification is done to ensure that the digestion is conducted under controlled heating (not boiling conditions) in a fume hood, with refluxing tubes or capped tubes to minimize volatilization losses of some minerals. Dried and homogenized tissue samples (0.4 g) were weighed into acid‐washed digestion vessels. The samples underwent microwave‐assisted digestion using a programmable microwave system (CEM MARS 6, CEM Corporation, USA). The digestion protocol consisted of a 15‐min pre‐digestion at room temperature, a 10‐min ramp to 120°C, and a 40‐min hold at 120°C. After cooling, the digests were filtered through Whatman No. 42 filter paper and diluted to a final volume of 50 mL with ultrapure water (18.2 MΩ·cm). Zn concentrations were measured using a flame atomic absorption spectrophotometer (AAnalyst 800, PerkinElmer, USA) with a Zn hollow cathode lamp. A five‐point calibration curve (0.1–2.0 mg L^−1^) was prepared using certified Zn standard solutions. Sample absorbance was measured in triplicate, with blank and quality control samples analyzed every 10 samples to ensure measurement stability. Zn concentrations in carrot tissues were expressed on a dry weight basis (mg kg^−1^ DW).

### 2.6 Statistical Analysis

Before analysis, data were tested for normality using the Shapiro–Wilk test and homogeneity of variance using Levene's test. The independence of observations was verified using the Durbin–Watson test. All statistical analyses were performed using R version 4.3 (R Core Team 2023). The effects of Zn application rate (0, 2, 4, and 6 kg ha^−1^), method of application (foliar and soil), and timing of application (30, 50, and 70 DAS) on tissue Zn concentration were analyzed using Type II analysis of variance (ANOVA), using Equation 1. When significant (*p* < 0.05) main effects or interactions were detected, means were compared using Tukey's HSD test.

(1) $$\text{Yijkl}=\mu +\mathrm{Mi}+\mathrm{Tj}+\mathrm{Rk}+\left(\mathrm{MT}\right)\mathrm{ij}+\left(\mathrm{MR}\right)\mathrm{ik}+\left(\mathrm{TR}\right)\mathrm{jk}+\left(\text{MTRijk}\right)+\text{Ɛijkl}$$

where: *Y*_*ijkl*_ is the observed response; *μ* is the overall mean; *M*_*i*_ is the effect of the *i* ^*th*^ application method; *T*_*j*_ is the effect of the *j* ^*th*^ application timing; *R*_*k*_ is the effect of the *k* ^*th*^ application rate; (*MT*)_*ij*_, (*MR*)_*ik*_, and (*TR*)_*jk*_ are the two‐way interaction effects; (*MTR*_*ijk*_) is a three‐way interaction effect; and *Ɛ*_*ijkl*_ is the random error term.

The relationship between Zn application rate and tissue Zn concentration was evaluated using linear and polynomial regression models. Model comparisons were performed using ANOVA, and the best‐fitting model was selected based on *R* ^2^ values and residual analysis. Estimated marginal means (EMMs) were calculated for significant interactions, and pairwise comparisons were performed with Tukey's adjustment for multiple comparisons. Treatment effects were expressed as percentage changes relative to control. Results are presented as means ± standard error (SE). Figures were created using the ggplot2 package in R, with error bars representing standard errors of the means.

Chlorophyll and plant height data were analyzed using a linear mixed‐effects model with the lme4 package in R. The model included fixed effects for Zn rate, application method, time, and their interactions, with week as a random effect to account for repeated measurements (model <− lmer(clo ~ rate * method * time + (1|week))). Here, ANOVA was performed using the car package to determine the significance of main effects and interactions. Estimated marginal means were computed using the emmeans package, and pairwise comparisons were conducted with Tukey's adjustment for multiple comparisons.

## 3 Results

### 3.1 Plant Growth and Chlorophyll Content of Leaves

**FIGURE 1:**
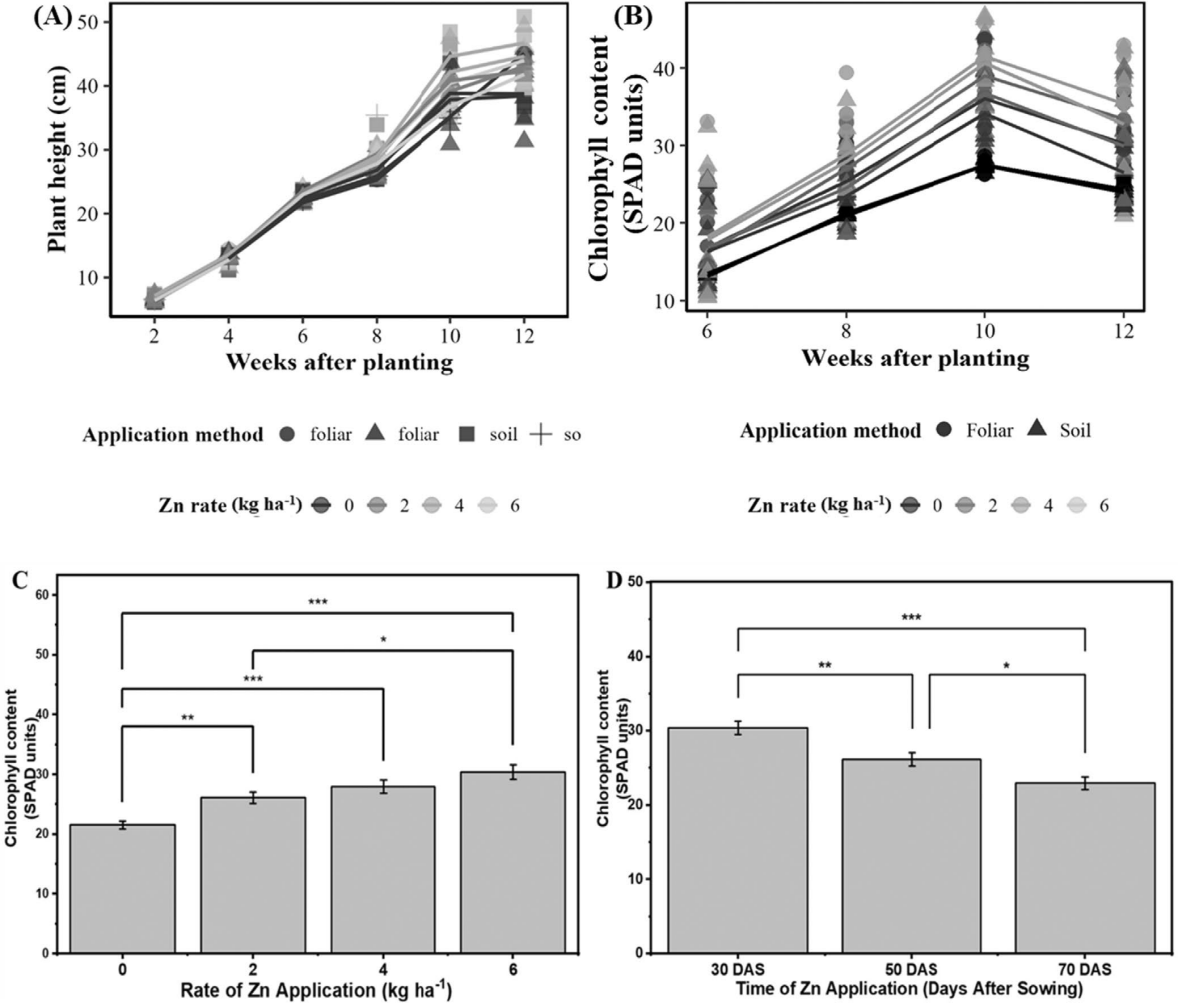
Plant height (A) and chlorophyll content (B) over time as affected by Zn application rate and method. Effect of Zn fertilization rate on chlorophyll content of pot‐grown carrot (C); effect of timing of Zn fertilization on chlorophyll content of pot‐grown carrot (D). Error bars represent standard errors of the means.

Zn application rate had a significant main effect (χ^2^ = 34.1857, df = 3, *p* < 0.001) on plant height. However, the main effects of the application method (χ^2^ = 0.6464, df = 3, *p* = 0.8857) and time (χ^2^ = 1.4984, df = 2, *p* = 0.4727) were not statistically significant. Figure 1A illustrates the changes in plant height over the growing period for different Zn rates and application methods. Plant height increased over time for all treatments, with the increase being more pronounced for higher Zn application rates, particularly from 4 to 10 WAP. The foliar application method resulted in slightly taller plants than soil application for the 4 and 6 kg/ha rates, but this difference was not statistically significant.

There were significant main effects of Zn application rate (χ^2^ = 190.28, df = 3, *p* < 0.001), time (df = 2, *p* = 0.026), and a significant interaction between rate and time (χ^2^ = 57.57, df = 6, *p* < 0.001) on chlorophyll content. The main effects of the application method and other interactions were not statistically significant (*p* > 0.05) (Figure 1B–D). Chlorophyll content increased by approximately 36% (from 21.55 to 29.35) as Zn application rates increased from 0 to 6 kg Zn ha^−1^ (Figure 1C). The highest chlorophyll content levels were observed at 30 days after sowing, followed by 50 and 70 days (Figure 1D). For plants which received Zn at 30 DAP, all Zn application rates significantly increased chlorophyll content compared to the control (0 kg/ha) for both foliar and soil applications (*p* < 0.001). The highest rate (6 kg/ha) resulted in the greatest increase in chlorophyll content. The effect of Zn application for plants receiving Zn at 50 DAP remained significant, but the differences between treatments decreased. For foliar application, all rates significantly increased chlorophyll content compared to the control (*p* < 0.001). Significant increases were observed for soil application for 4 and 6 kg/ha rates (p < 0.001). The effect of Zn application was less pronounced for plants which received Zn at 70 DAP. Only the 4 and 6 kg/ha rates for foliar application maintained significantly higher chlorophyll content than the control (*p* < 0.05). For soil application, no significant differences were observed between treatments (*p* > 0.05) (Figure 1B).

### 3.2 Yield and Yield‐Dependent Parameters

**FIGURE 2:**
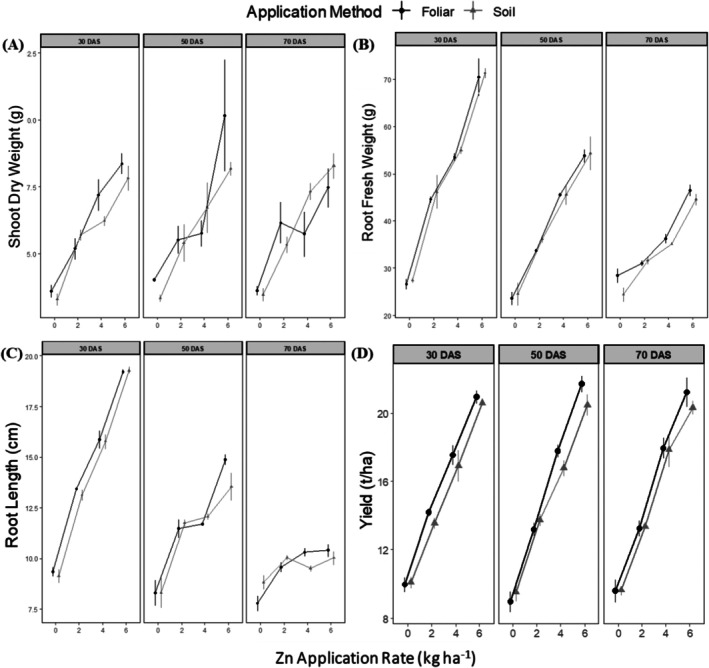
Effect of Zn fertilization rate, time and method of application on shoot dry weight (A), root fresh weight (B), root length (C), and yield (D) of pot‐grown carrot. Error bars represent standard errors of the means.

There was a significant main effect of Zn application rate on shoot dry weight (F(3, 48) = 11.04, *p* < 0.001) and root fresh weight (F(3, 48) = 118.31, *p* < 0.001) (Figure 2A,B). Compared to the control, Zn application at 2, 4, and 6 kg/ha resulted in SDW increases of 1.49–2.54 g (18.9%–32.2% increase), 1.73–3.84 g (22.0%–48.7% increase), and 3.85–6.13 g (48.8%–77.8% increase), respectively (all *p* < 0.05) (Figure 2A). Neither the method of Zn application (F(1, 48) = 0.12, *p* = 0.73) nor the timing of application (F(2, 48) = 0.14, *p* = 0.87) showed significant main effects on SDW. No significant interactions were detected among the factors studied. Compared to the control, Zn application at 2, 4, and 6 kg/ha increased RFW by 2.61–18.76 g (8.7%–62.5% increase), 7.89–27.58 g (26.3%–92.0% increase), and 18.07–43.94 g (60.2%–146.5% increase), respectively (all *p* < 0.05) (Figure 2B). A significant interaction between rate and time of application was found (F(6, 48) = 11.29, *p* < 0.001), with the effect of Zn rate being more pronounced at earlier application times. The method of Zn application (F(1, 48) = 0.13, *p* = 0.72) and timing of application (F(2, 48) = 2.11, *p* = 0.13) did not show significant main effects on RFW.

For the length of the roots, there were significant main effects of both Zn application rate (F(3, 48) = 142.43, *p* < 0.001) and timing of application (F(2, 48) = 5.30, *p* < 0.01). Zn application at 2, 4, and 6 kg/ha increased RL by 1.21–4.07 cm (11.5%–38.8% increase), 0.69–6.64 cm (6.6%–63.2% increase), and 1.21–10.15 cm (11.5%–96.7% increase), respectively, compared to the control (all *p* < 0.05, except for some comparisons at 70 DAS) (Figure 2C). A significant interaction between rate and time of application was observed (F(6, 48) = 20.40, *p* < 0.001), with the effect of Zn rate being more pronounced at earlier application times (Figure 2D). The method of Zn application did not significantly affect root length (F(1, 48) = 0.19, *p* = 0.66).

There was a significant main effect of Zn application rate on carrot yield (F(3, 48) = 80.36, *p* < 0.001), with significant yield increases with each increment in Zn application rate (Figure 2D). Compared to the control, Zn application at 2, 4, and 6 kg/ha resulted in yield increases of 3.47–4.23 t/ha (32.1% or 1.32‐fold increase), 6.83–8.83 t/ha (63.2% or 1.63‐fold increase), and 10.50–12.77 t/ha (97.2% or 1.97‐fold increase), respectively (all *p* < 0.001). Furthermore, significant yield differences were observed between all Zn application rates, with higher rates consistently producing greater yields (Figure 2D). Neither the method of Zn application (foliar vs. soil) nor the timing of application (30, 50, or 70 days after sowing) showed significant main effects on carrot yield (F(1, 48) = 0.03, *p* = 0.86 and F(2, 48) = 0.93, *p* = 0.40, respectively). No significant interactions were detected among the factors studied, including rate and method (F(3, 48) = 0.24, *p* = 0.87), rate and time (F(6, 48) = 0.84, *p* = 0.54), method and time (F(2, 48) = 0.15, *p* = 0.86), or the three‐way interaction of rate, method, and time (F(6, 48) = 0.49, *p* = 0.81).

### 3.3 Tissue Zn Concentration

#### 3.3.1 Shoot Zn Concentration

**FIGURE 3:**
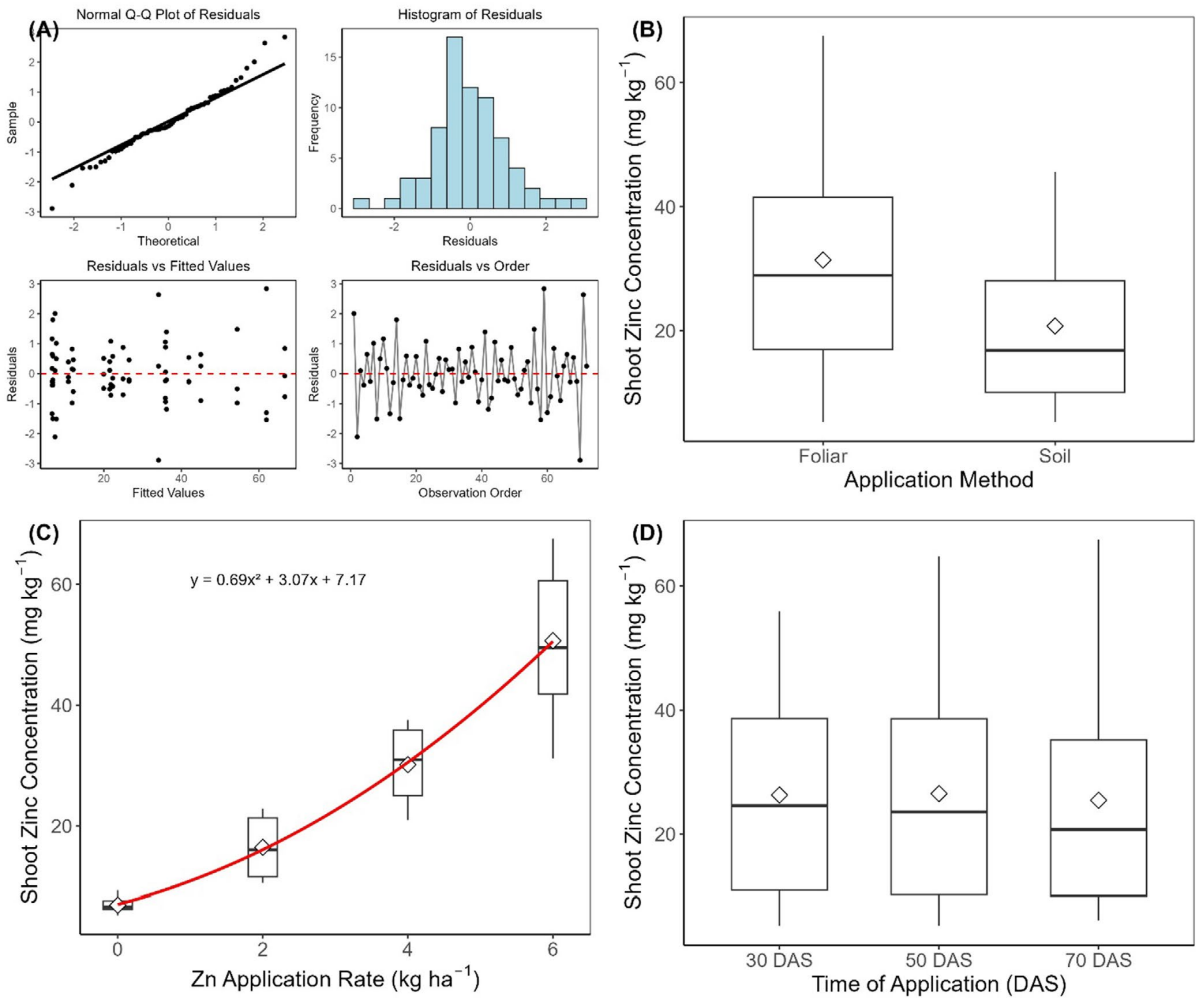
Shoot Zn concentration of carrot plants grown in soil‐filled pots. Diagnostic plots were used to verify normality assumptions (A), effect of method (B), rate (C), and time (D) of Zn application. Figure 3C also shows the relationship between Zn application rate and shoot Zn concentration in carrots. The boxplots show the data distribution at each rate, where the horizontal line represents the median, the box represents the interquartile range (IQR), and whiskers extend to 1.5 × IQR. White diamonds indicate means. The red line represents the fitted quadratic regression (y = ax^2^ + bx + c, R^2^ = 0.85, *p* < 0.001).

The Shapiro–Wilk test confirmed the normal distribution of residuals (W = 0.945, *p* = 0.313), while Levene's test verified homoscedasticity (F = 0.935, *p* = 0.798). The Durbin–Watson test (DW = 2.631, *p* = 0.36) confirmed the independence of observations (Figure 3A). There were highly significant effects of application method (F(1, 48) = 1392.99, *p* < 0.001; Figure 3B), rate (F(3, 48) = 4414.04, *p* < 0.001; Figure 3C), and time of application showed a significant main effect (F(2, 48) = 5.24, *p* = 0.009; Figure 3D). Mean Zn concentrations were similar at 30 DAS (26.3 ± 3.46 mg kg^−1^) and 50 DAS (26.5 ± 3.77 mg kg^−1^), with a slight decrease at 70 DAS (25.4 ± 3.91 mg kg^−1^). Foliar application resulted in markedly higher shoot Zn concentrations (31.4 ± 3.39 mg kg^−1^) than soil application (20.8 ± 2.24 mg kg^−1^). Zn concentration increased nonlinearly with application rate, following a quadratic relationship (R^2^ = 0.847) rather than a linear response (R^2^ = 0.823). Compared to the control (7.04 ± 0.29 mg kg^−1^), Zn concentrations increased by 134% at 2 kg ha^−1^, 328% at 4 kg ha^−1^, and 620% at 6 kg ha^−1^ (Figure 3C).

**FIGURE 4:**
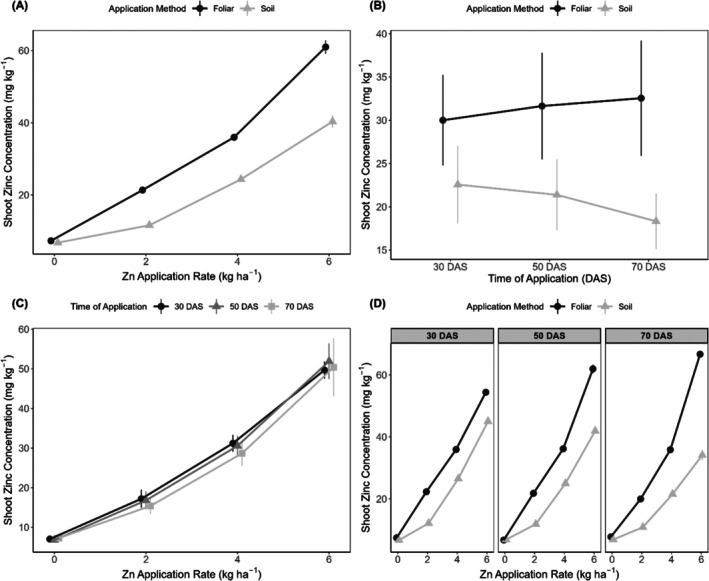
Shoot Zn concentration of carrot plants grown in soil‐filled pots. Two‐way interactive effects: (A) application rate x method of application, (B) method x time of application, (C) application rate x time of application, and (D) rate × method × time of application. Error bars represent standard errors of the means.

There were highly significant interactive effects of method and rate (F(3, 48) = 209.87, *p* < 0.001) on shoot Zn concentration (Figure 4A). Time of application interacted significantly with both method (F(2, 48) = 47.72, *p* < 0.001) and rate (F(6, 48) = 3.70, *p* = 0.004; Figure 4B). The foliar application difference became more pronounced with increasing Zn rates, reaching maximum divergence at 6 kg ha^−1^ (60.99 vs. 40.34 mg kg^−1^ for foliar and soil applications, respectively). The method × time interaction showed that the superiority of foliar application intensified over time, with the difference between methods increasing from 7.43 mg kg^−1^ at 30 DAS to 14.22 mg kg^−1^ at 70 DAS. The rate × time interaction showed that Zn accumulation was most pronounced at 50 DAS for the highest rate (51.92 ± 0.49 mg kg^−1^; Figure 4C), while the response to lower rates remained relatively consistent across application times. While foliar and soil applications showed similar shoot Zn concentrations at the control rate (7.29 vs. 6.78 mg kg^−1^, *p* = 0.376), the difference between methods increased markedly with increasing Zn rates. At 2 kg ha^−1^, foliar application resulted in 84% higher Zn concentration than soil application (21.35 vs. 11.60 mg kg^−1^). This difference widened further at 4 kg ha^−1^ (35.98 vs. 24.33 mg kg^−1^) and reached maximum divergence at 6 kg ha^−1^, where foliar application achieved 51% higher concentration than soil application (60.99 vs. 40.34 mg kg^−1^). The significant three‐way interaction (F(6, 48) = 32.45, *p* < 0.001) indicated that the method × rate relationship varied across application times (Figure 4D). The superiority of foliar application over soil application became more pronounced at later application times, particularly at higher Zn rates. At 70 DAS, the difference between foliar and soil methods was greatest (14.22 mg kg^−1^), while at 30 DAS, the difference was smaller (7.43 mg kg^−1^). This temporal pattern was most evident at the 6 kg ha^−1^ rate, where foliar application maintained consistently high Zn concentrations across all timings. In contrast, soil application showed declining effectiveness at later application times (Figure 4).

#### 3.3.2 Root Zn Concentration

**FIGURE 5:**
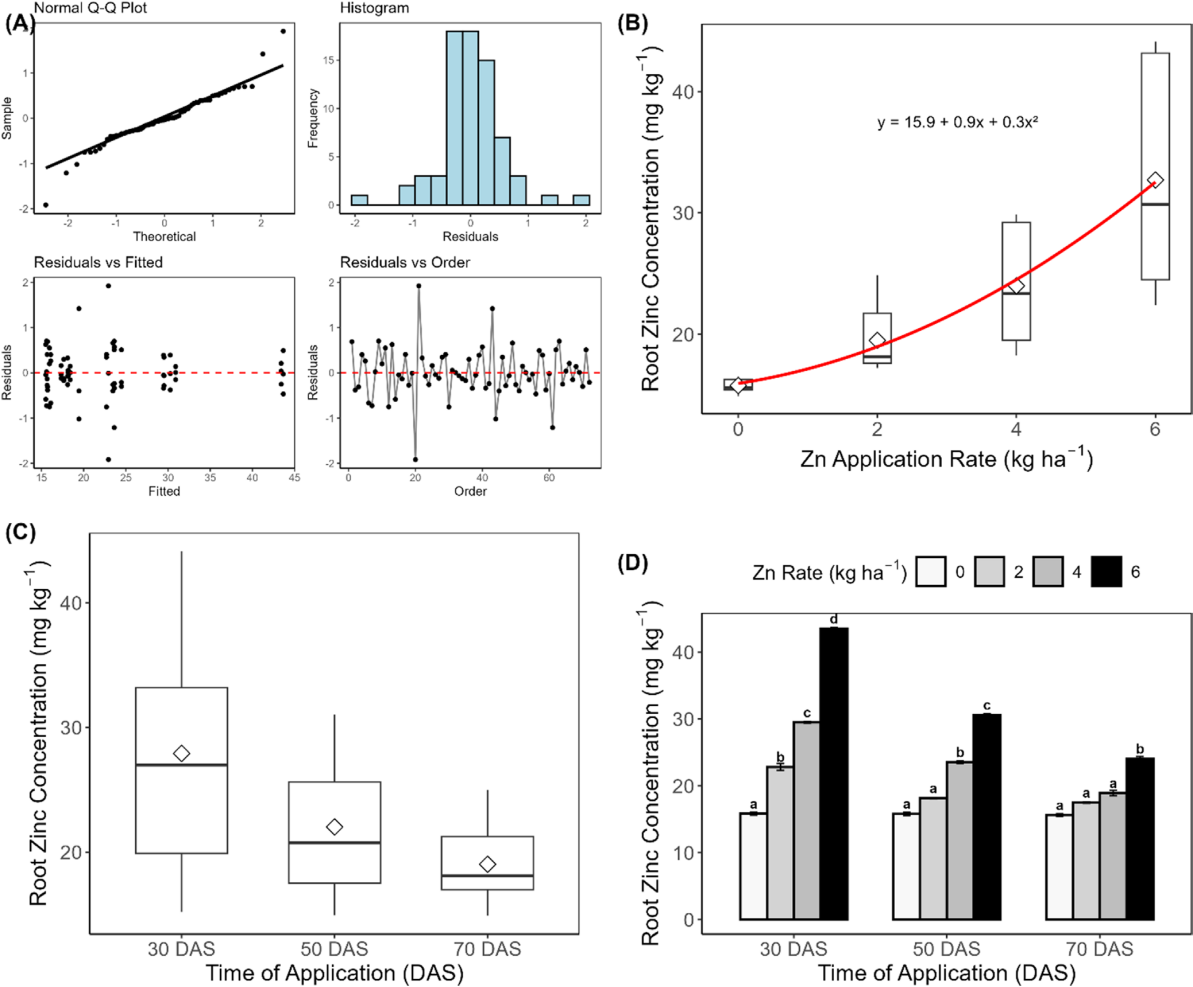
Root Zn concentration of carrot plants grown in soil‐filled pots. Diagnostic plots were used to verify normality assumptions (A), the effect of rate (B), and time (C) of Zn application. Figure 5B also shows the relationship between Zn application rate and root Zn concentration in carrots. The boxplots show the data distribution at each rate, where the horizontal line represents the median, the box represents the interquartile range (IQR), and the whiskers extend to 1.5 × IQR. White diamonds indicate means. The red line represents the fitted quadratic regression (y = ax^2^ + bx + c, R^2^ = 0.85, *p* < 0.001). Figure 5D shows the two‐way interactive effect of application rate x time of application.

The Shapiro–Wilk test indicated a slight deviation from normality (W = 0.945, *p* = 0.00355). However, this was not considered problematic given the large sample size (*n* = 72) and ANOVA's robustness to minor normality violations. Homoscedasticity was confirmed by Levene's test (F = 0.935, *p* = 0.556), indicating equal variances across treatment groups. The Durbin–Watson test (DW = 2.631, *p* = 0.966) verified the independence of observations. No influential observations or outliers were detected in the model diagnostics, supporting the validity of the subsequent ANOVA analyses (Figure 5A).

There were significant main effects of Zn application rate (F(3, 48) = 2162.30, *p* < 0.001; Figure 5C) and timing of application (F(2, 48) = 1105.79, *p* < 0.001; Figure 5C) on root Zn concentration. The relationship between Zn application rate and root Zn concentration followed a quadratic trend rather than a linear response. The polynomial regression model (*R* ^2^ = 0.85, *p* < 0.01) provided a significantly better fit than the linear model (R^2^ = 0.82). This nonlinear response was particularly evident in the early application timing (30 DAS), where the increase in root Zn concentration was most pronounced between 4 and 6 kg ha^−1^, followed by a gradual plateauing effect (Figure 5B).

A significant interaction between rate and time was also observed (F(6, 48) = 237.96, *p* < 0.001; Figure 5D). The method of Zn application showed no significant effect (F(1, 48) = 0.001, *p* = 0.971), and no other interactions were significant. Root Zn concentration increased significantly with each increment in Zn application rate. Compared to the control (15.77 ± 0.16 mg kg^−1^), Zn application at 2, 4, and 6 kg ha^−1^ increased root Zn concentration by 23.6% (19.49 ± 0.16 mg kg^−1^), 52.2% (24.00 ± 0.16 mg kg^−1^), and 107.5% (32.72 ± 0.16 mg kg^−1^), respectively (all *p* < 0.001). The rate × time interaction suggested that Zn accumulation in roots was most pronounced at early application (30 DAS). At this time, root Zn concentrations increased from 15.8 ± 0.27 mg kg^−1^ in the control to 43.5 ± 0.27 mg kg^−1^ at 6 kg ha^−1^ (175% increase). The magnitude of response diminished with later application times. At 50 DAS, the highest rate increased root Zn to 30.6 ± 0.27 mg kg^−1^ (93.7% increase), while at 70 DAS, the increase was more modest, reaching 24.1 ± 0.27 mg kg^−1^ (54.5% increase). All pairwise comparisons between rates within each application time were significant (*p* < 0.001), with distinct groupings (a–d) indicating clear rate–response relationships at each time. The method of Zn application showed no significant effect (F(1, 48) = 0.001, *p* = 0.971), and no significant interactions were observed between method and rate (F(3, 48) = 1.12, *p* = 0.352) or method and time (F(2, 48) = 0.03, *p* = 0.968). Similarly, the three‐way interaction between method, rate, and time was insignificant (F(6, 48) = 1.03, *p* = 0.416).

## 4 Discussion

### 4.1 Zn Application Effects on Growth and Photosynthetic Parameters

Our findings demonstrate that Zn fertilization significantly enhanced carrot growth parameters, with the response magnitude varying by application rate and timing. The 36% increase in chlorophyll content with increasing Zn rates (up to 6 kg ha^−1^) (Figure 1B) aligns with Zn's crucial role in chlorophyll synthesis and photosynthetic efficiency (Cakmak 2008). This substantial improvement in chlorophyll content parallels findings by Adil et al. (2022), Aslam et al. (2014), and Tayyeba et al. (2013), who reported similar enhancements in chlorophyll synthesis with Zn supplementation, reflecting Zn's essential role in chlorophyll formation and protein synthesis. The more pronounced effect of early Zn application (30 DAS) on chlorophyll content suggests that adequate Zn supply during initial growth stages is critical for establishing robust photosynthetic machinery. This timing‐dependent response may be attributed to Zn's involvement in carbonic anhydrase activity and chloroplast development (Escudero‐Almanza et al. 2012). The absence of significant differences between foliar and soil application methods on plant height and chlorophyll content contrasts with previous studies showing superior results with foliar application in other crops (Praharaj et al. 2021). This disparity might be explained by carrots' extensive root systems and efficient nutrient uptake mechanisms, which allow effective Zn acquisition through both application methods.

### 4.2 Impact on Yield Components and Root Morphology

The substantial yield improvements observed with Zn application (up to 97.2% increase at 6 kg ha^−1^) (Figure 2D) are important for carrot production systems. This enhanced response might be attributed to carrots' extensive root systems, facilitating better nutrient uptake. The quadratic relationship between Zn rate and yield parameters indicates an optimum application range beyond which additional Zn might not provide proportional benefits. Numerous studies have shown that carrots, maize, and wheat exhibit a positive yield response to Zn fertilization (Awad et al. 2021; Palai et al. 2020). Awad et al. (2021) reported that carrot yields increase by over 85% with foliar Zn fertilization. With soil Zn fertilization, Liu et al. (2020) also observed maize yield improvements ranging from 4% to 17%.

The significant interaction between rate and timing for root fresh weight and length (Figure 2) reveals the importance of synchronized nutrient supply with critical growth stages. Early application (30 DAS) proved most effective, likely due to Zn's role in root development and cellular elongation during the initial growth phase (Bhatt et al. 2020). This timing advantage diminished with later applications, possibly highlighting the limited window for optimizing Zn's impact on root development. The results corroborate previous findings in wheat, where higher yield was recorded when Zn was applied at the stem elongation and tillering stage compared to when applied at the milking stage (El‐Dahshouri 2017). In contrast, Boonchuay et al. (2013) reported that Zn application at different rice growth stages did not significantly impact the grain yield.

### 4.3 Differential Zn Accumulation Patterns in Shoot and Root Tissues

Foliar Zn application has been reported to be more effective in improving crop productivity than soil application (Xue et al. 2023). In potato biofortification studies, foliar Zn application increased tuber Zn concentrations by 170%–284% over control, while soil application achieved only 56%–116% increases (Ahammed et al. 2025). Similarly, wheat studies showed foliar Zn application increased grain Zn concentration by 61% with improved bioavailability. In comparison, soil application had no significant effect on grain Zn despite increasing soil DTPA‐Zn by 174% (Zhao et al. 2014).

The contrasting responses of shoot and root Zn concentrations to application methods observed in the present study are noteworthy and reflect well‐established physiological mechanisms governing Zn transport in plants. While foliar application resulted in significantly higher shoot Zn concentrations (31.4 vs. 20.8 mg kg^−1^), root Zn concentrations showed no significant difference between application methods. This pattern suggests distinct Zn translocation mechanisms between aerial and underground carrot tissues. However, root concentrations show minimal differences between application methods, as demonstrated in maize, where both soil and foliar applications effectively increased root Zn concentrations (Vasconcelos et al. 2011). This pattern reflects Zn's preferential translocation to shoots when applied foliarly versus a more uniform distribution with soil application.

The markedly higher shoot Zn concentrations achieved through foliar application (51% higher than soil application at 6 kg ha^−1^) suggest more efficient leaf uptake, possibly due to direct absorption and translocation through the phloem. Other reports have suggested that the superior performance of foliar application is attributed to Zn's phloem mobility, allowing direct transport from leaves to developing reproductive organs (Ahammed et al., 2025; Haslett et al. 2001). Buturi et al. (2023) reported an over 94% rise in root Zn levels of carrots when Zn‐EDTA was applied to the foliage. Foliar application‐based biofortification programs typically benefit from the increased phloem mobility of minerals, driven by chelating compounds like sugars and other organic metabolites. These substances promote the efficient translocation of minerals from the leaves to growing sink organs such as roots, fruits, and grains (Gupta et al. 2016). Foliar application offers several benefits, such as preventing Zn fixation and avoiding the impact of antagonistic nutrients on Zn uptake, among others (Prasad et al. 2014). Thus, foliar Zn fertilization allows for efficient absorption and transportation via the phloem, as evidenced in wheat studies using radio‐labeled Zn (65Zn), especially under conditions of low Zn availability (Erenoglu et al. 2002; Haslett et al. 2001).

However, the absence of significant differences between application methods in root Zn concentration observed here suggests that while foliar application may be more effective for shoot enrichment, both methods can equally achieve target root Zn levels. The absence of significant differences between application methods in root Zn concentration might also reflect physiological constraints to Zn movement from leaves to storage roots. Zn translocation from leaves to storage roots in crop plants can be constrained by several physiological mechanisms, with root vacuoles, for example, acting as symplastic checkpoints that sequester Zn and limit its movement to shoots and storage tissues (Ricachenevsky et al. 2018). The low mobility of Zn in the phloem might also represent a major barrier, restricting Zn accumulation in storage organs such as tubers, fruits, and seeds, where concentrations rarely exceed 30–100 mg kg^−1^ dry matter (White and Broadley 2011). Substantial foliar‐applied Zn can become sequestered in leaf cell walls, vacuoles, or bound to metalloproteins, thereby limiting its redistribution to underground organs (Broadley et al. 2007; Broadley et al. 2012). Storage roots such as carrot taproots act primarily as carbohydrate sinks rather than strong Zn sinks, unlike seeds or grains, where biofortification studies typically report large translocation benefits from foliar feeding (Cakmak and Kutman 2018). Additionally, while foliar feeding enhanced shoot Zn due to direct uptake, the storage root Zn was governed more by root uptake mechanisms, explaining the similar root Zn concentrations regardless of application method.

### 4.4 Temporal Dynamics of Zn Uptake and Accumulation

The significant interaction between application timing and Zn accumulation patterns provides crucial insights into optimal biofortification strategies. Early application (30 DAS) proved most effective for root Zn accumulation, with concentrations increasing by 175% compared to the control (Figure 5C,D). This advantage diminished progressively at 50 DAS (93.7% increase) and 70 DAS (54.5% increase), suggesting a critical window for Zn intervention in carrot development. The greater effectiveness of early Zn application for root accumulation reflects the synchronization of nutrient availability with peak root sink strength and physiological activity. At early developmental stages, carrot roots are rapidly expanding, with high activity of Zn uptake systems such as ZIP family transporters that enhance Zn influx into cortical and vascular tissues (Gupta et al. 2016). Younger roots possess less suberization and lignification in the endodermis, facilitating apoplastic movement of Zn into the stele compared to mature roots (Marschner 2011). Early Zn application generally promotes better root development, with soil Zn fertilization significantly increasing root dry weight, root length density, and root surface area in winter wheat (Liu et al. 2020). In sandy soils with low cation exchange capacity, such as those in this study (sandy loam with CEC of 3.65 cmol kg^−1^), early application minimizes Zn losses due to leaching, further enhancing root uptake efficiency (Alloway 2008).

These findings contrast with cereal crops, where later applications prove more effective. Increased grain Zn in wheat was observed when foliar Zn was applied after flowering compared to before flowering (Cakmak et al. 2010; Ozturk et al. 2006), while brown rice Zn concentration increased 56% with Zn fertilization after flowering (Boonchuay et al. 2013). These differences reflect species‐dependent mechanisms for Zn uptake and the distinct physiological priorities of storage roots versus reproductive tissues. The quadratic relationship between application rate and Zn accumulation (*R* ^2^ = 0.847; Figure 5B) indicates a physiological ceiling for Zn uptake, vital for optimizing biofortification protocols. This non‐linear response suggests complex uptake and translocation mechanisms, possibly involving homeostatic regulation of Zn transport to storage tissues.

### 4.5 Implications for Biofortification Strategies

Our results provide several practical implications for Zn biofortification in carrots. The superior performance of foliar application for shoot Zn enrichment, coupled with the timing‐dependent root accumulation patterns, suggests that a combined approach might be most effective for whole‐plant Zn enhancement. The timing of Zn application was a critical factor, with early application (30 DAS) consistently showing superior results across multiple parameters. Also, the nonlinear response to Zn application rates indicates that optimal rates might vary depending on target tissues and desired outcomes. The consistent yield and Zn concentration improvements up to 6 kg ha^−1^ suggest that this rate might serve as a practical upper limit for biofortification programs under similar conditions, balancing enrichment goals with economic considerations.

The soil properties in our study, particularly the slightly acidic pH (5.77), low organic matter content (1.48%), and sandy loam texture (78.39% sand), are representative of many tropical arable soils, especially in the coastal savannah agroecology of Ghana. These soil characteristics likely influenced the observed Zn dynamics in several ways. The predominantly sandy texture, combined with low organic matter content, suggests limited Zn retention capacity, which may explain the high efficiency of early Zn applications (30 DAS) compared to later timings. The slightly acidic pH falls within the optimal range for Zn availability (5.5–6.5), potentially contributing to the substantial yield responses observed (up to 97.2% increase) despite the relatively low initial soil Zn content (2.19 μg/g).

The broader implications for tropical agriculture are significant. Given that many soils from tropical regions share similar characteristics, being highly weathered, acidic, and low in organic matter (Adekiya et al. 2023; Soares and Alleoni 2008), our findings suggest that Zn biofortification strategies could be particularly effective in these regions. The low cation exchange capacity (3.65 cmol kg^−1^) observed in our study is typical of soils from tropical regions (da Costa et al. 2020; Lucena et al. 2024) and indicates that split applications or foliar supplementation might be more effective than single soil applications for long‐term Zn availability. Moreover, the strong response to Zn application despite the challenging soil conditions suggests that biofortification could be a viable strategy even in resource‐poor regions with similar soil constraints.

For the coastal savannah agroecology and similar tropical regions, our results indicate that early Zn intervention is crucial, particularly given the rapid nutrient leaching potential in sandy soils, and the combination of soil and foliar applications might be most effective in overcoming these soils' limited nutrient retention capacity. The observed quadratic response to Zn application suggests that even moderate rates could achieve significant benefits, potentially making biofortification economically viable for resource‐limited farmers. The substantial yield increases demonstrate that Zn biofortification can simultaneously address nutritional goals and agricultural productivity in tropical regions. However, detailed cost–benefit analyses will be essential to confirm economic feasibility. These findings are particularly relevant for sub‐Saharan Africa, where similar soil conditions are widespread, and Zn deficiency affects soil fertility and human nutrition. The effectiveness of Zn biofortification under these challenging soil conditions provides a promising pathway for addressing agricultural productivity and nutritional security in tropical regions.

Ultimately, the practical implications of our results for farmers are enormous. The identified optimal Zn application threshold of 6 kg ha^−1^ provides a practical baseline for carrot biofortification programs, though site‐specific adjustments will be necessary. Farmers can adapt this rate based on initial soil Zn testing, with lower rates potentially sufficient in soils with higher baseline Zn content or better nutrient retention capacity than our study's very low CEC conditions (3.65 cmol kg^−1^). The quadratic response pattern suggests that exceeding optimal rates provides diminishing returns, making precise application economically important for resource‐limited farmers. Accordingly, extension services should prioritize soil testing protocols that include available Zn content, CEC, and texture analysis to guide rate adjustments. Additionally, the superior performance of early application timing (30 DAS) combined with the identified optimal rate provides a protocol that can be adapted across similar agroecological zones, with local validation trials recommended to fine‐tune recommendations for specific soil‐climate combinations.

### 4.6 Study Limitations and Future Research Directions

While our study provides valuable data on Zn biofortification in carrots, some aspects warrant further investigation. Understanding Zn speciation in different plant tissues and its implications for bioavailability remains a critical research gap that could inform more targeted biofortification strategies. Field validation under various soil conditions and environmental factors would strengthen the practical applicability of our findings, as pot experiments may not fully reflect the complexities of field situations. Future studies should also examine potential interactions between Zn and other nutrients and the possible effects of anti‐nutritional factors on Zn bioavailability. The stability of accumulated Zn during storage and processing requires investigation to ensure the sustained nutritional value of biofortified carrots throughout the food supply chain. From a practical implementation perspective, economic analyses of different application strategies, including cost–benefit assessments and factors affecting farmer adoption, would contribute to developing more feasible biofortification protocols. While our results suggest potential economic benefits through increased yields and moderate Zn application rates, detailed assessments of input costs, labor requirements, application equipment, storage and processing considerations, and potential market premiums for biofortified produce are essential for developing economically viable implementation strategies.

This study was conducted using a single carrot variety, which limits the generalizability of our findings across different genetic backgrounds. Research on Zn uptake efficiency across different cultivars and soil conditions reveals significant variability in plant responses. In potato cultivars, Zn efficiency varied substantially, with Kufri Badshah achieving 102% relative yield under low Zn conditions compared to Kufri Chandramukhi's 71%, primarily due to differences in root–shoot ratios (Trehan and Sharma 2003). Similarly, maize cultivars showed Zn efficiency ranging from 62.3% to 81.2% under Zn‐deficient calcareous soil conditions (Chaab et al. 2011). Carrot cultivars may also exhibit genetic diversity that may influence Zn uptake efficiency, root‐to‐shoot translocation, and overall biofortification potential. The variety used in this study demonstrated clear responsiveness to Zn treatments, but the magnitude of response may vary substantially among different carrot cultivars. Multi‐variety screening studies will be essential to identify the most promising genetic backgrounds for Zn biofortification programs and to develop variety‐specific application protocols. Future research should prioritize testing diverse carrot genotypes to establish the range of Zn biofortification potential across commercially important varieties. These research directions would collectively enhance our understanding of Zn biofortification in carrots and support the development of practical protocols, particularly for regions with Zn‐deficient soils.

## 5 Conclusion

This study provides insights into optimizing Zn biofortification strategies for carrots. Zn application significantly enhanced growth parameters, yield, and tissue Zn concentrations, with the magnitude of improvement varying by application method, rate, and timing. Foliar application proved more effective for shoot Zn enrichment, while early soil applications (30 DAS) were critical for maximizing root Zn accumulation. The observed quadratic response to Zn rates indicates an optimal threshold (6 kg ha^−1^) beyond which additional inputs may yield diminishing returns.

Our findings underscore the importance of tailoring biofortification protocols to crop‐specific nutrient uptake dynamics and soil conditions. Combining soil and foliar applications offers a practical approach to overcoming challenges for tropical regions characterized by sandy loam soils with low organic matter content and limited nutrient retention capacity. The substantial yield increases demonstrate that Zn biofortification can simultaneously address agricultural productivity and nutritional security goals.

Future research should focus on field validation under diverse agroecological conditions to confirm these results at scale. Additionally, exploring the interactions between Zn and other nutrients and the bioavailability of accumulated Zn during storage and processing will further refine biofortification strategies. This study highlights the potential of carrot biofortification as a sustainable intervention to combat micronutrient malnutrition in resource‐limited areas.

## Conflicts of Interest

The authors declare no conflicts of interest.

## Data Availability

The data that support the findings of this study are available in this article.
